# Differences in ovarian aging patterns between races are associated with ovarian genotypes and sub-genotypes of the *FMR1* gene

**DOI:** 10.1186/1477-7827-10-77

**Published:** 2012-09-10

**Authors:** Norbert Gleicher, Ann Kim, Andrea Weghofer, David H Barad

**Affiliations:** 1 The Center for Human Reproduction (CHR), New York, NY, USA; 2 Foundation for Reproductive Medicine, New York, NY, USA; 3 Department of Obstetrics and Gynecology, Medical University Vienna, Vienna, Austria

**Keywords:** Ovarian reserve, Ovarian aging, Follicle stimulating hormone, Anti-Müllerian hormone, Oocyte yield, *FMR1* gene, Infertility, In vitro fertilization

## Abstract

**Background:**

Ovarian aging patterns differ between races, and appear to affect fertility treatment outcomes. What causes these differences is, however, unknown. Variations in ovarian aging patterns have recently been associated with specific ovarian genotypes and sub-genotypes of the *FMR1* gene. We, therefore, attempted to determine differences in how functional ovarian reserve (FOR) changes with advancing age between races, and whether changes are associated with differences in distribution of ovarian genotypes and sub-genotypes of the *FMR1* gene.

**Methods:**

We determined in association with in vitro fertilization (IVF) FOR in 62 young Caucasian, African and Asian oocyte donors and 536 older infertility patients of all three races, based on follicle stimulating hormone (FSH), anti-Müllerian hormone (AMH) and oocyte yields, and investigated whether differences between races are associated with differences in distribution of *FMR1* genotypes and sub-genotypes.

**Results:**

Changes in distribution of mean FSH, AMH and oocyte yields between young donors and older infertility patients were significant (all P < 0.001). Donors did not demonstrate significant differences between races in AMH and FSH but demonstrated significant differences in oocyte yields [F(2,59) = 4.22, P = 0.019]: Specifically, African donors demonstrated larger oocyte yields than Caucasians (P = 0.008) and Asians (P = 0.022). In patients, AMH levels differed significantly between races [F (2,533) = 4.25, P = 0.015]. Holm-Sidak post-hoc comparisons demonstrated that Caucasians demonstrated lower AMH in comparison to Asians (P = 0.007). Percentages of *FMR1* genotypes and sub-genotypes in patients varied significantly between races, with Asians demonstrating fewer *het-norm/low* sub-genotypes than Caucasians and Africans (P = 0.012).

**Conclusion:**

FOR changes in different races at different rates, and appears to parallel ovarian *FMR1* genotypes and sub-genotype distributions. Differences in ovarian aging between races may, therefore, be *FMR1*-associated.

## Background

Evidence has accumulated over the last decade that ovarian aging is genetically controlled [1,2], and that significant differences can be observed between races/ethnicities [3]. These differences also appear reflected in varying infertility treatment outcomes between races [4,5], though some investigators have disputed such differences [6].

We recently described ovarian genotypes and sub-genotypes of the *FMR1* gene, which have been associated with varying ovarian aging patterns [7,8] as well as differences in pregnancy chances with in vitro fertilization (IVF). They are based on a normal range of CGG triple nucleotide repeats (CGG_n_) of 26 to 34, with median of 30, allowing for the determination of genotypes and sub-genotypes of *FMR1*, distinct from traditional genotypes, which are primarily used to assess neuro-psychiatric risks [3,8].

In a study of 339 infertility patients we previously demonstrated that different races were associated with different distributions of *FMR1* genotypes and sub-genotypes, and that genotypes and sub-genotypes in different races were associated with varying IVF pregnancy chances [3].

It now occurred to us that differences in ovarian aging between races/ethnicities should be reflected in cross-sectional observations of functional ovarian reserve (FOR) over time. FOR represents the part of total ovarian reserve that, to somewhat varying degrees, can be evaluated by follicle stimulating hormone (FSH), anti-Müllerian hormone (AMH), and oocyte yields in association with in vitro fertilization (IVF), and has been equated with “ovarian age” [9].

Consequently, if different races age ovaries differently, they over time should demonstrate divergent FOR. In an attempt to further clarify what influences variations between races in ovarian aging patterns, we in this study present such a cross-sectional study over time, by comparing FOR in young oocyte donors and older infertility patients of different races, and by associating their FOR changes with *FMR1* genotype and sub-genotype distributions.

## Methods

This study assessed 62 first oocyte donation cycles in young oocyte donors and 536 first IVF cycles in infertility patients. The study, thus, compared cross-sectionally a young egg donor population with a significantly more aged patient population. Individuals in both patient groups were classified by race/ethnicity in accordance with NIH guidelines [10] as Caucasians, Africans and Asians. Asian patients almost universally were of Chinese descent. Because Hispanic patients often cross racial/ethnic identities and are under NIH guidelines, therefore, defined as “ethnicity” rather than race, they were excluded from consideration, as were other patients of mixed racial/ethnic backgrounds, unless they self-identified as belonging to one of above three specific races.

As reflection of FOR, we for all patients assessed FSH, AMH prior to cycle start, and oocyte yield at time of oocyte retrieval. FSH and AMH were assessed by routine commercial assays.

All oocyte retrievals were performed by two of the authors (N.G, D.H.B.), who in over eight years of continuous quality control never differed in number of oocytes obtained during oocyte retrievals. Variations in oocyte numbers retrieved between physicians have been suggested a limiting factor in using oocyte yields as the most accurate representation of FOR [11].

Here presented patient populations lend themselves well to comparisons since both groups received homogenous treatments. Oocyte donors received ovarian stimulation with a long agonist protocol and, depending on age and AMH, between 150 and 300 IU of gonadotropins in form of human menopausal gonadotropin (hMG). The center’s patient population, as is apparent from FSH and AMH levels (Table 1), in approximately half of all patients carried a primary diagnosis of diminished ovarian reserve (DOR), characterized by either and/or abnormally high FSH or low AMH levels, as previously defined [12,13]. The real prevalence of DOR was, however, even higher, as many patients carried DOR as a secondary diagnosis. The center’s current patient population undergoing IVF cycles is in approximately 90 percent afflicted by DOR.

**Table 1:** Patient characteristics in oocyte donors and infertility patients

	**Oocyte donors**				**Infertile patients**			
	**Total**	**Caucasian**	**African**	**Asian**	**Total**	**Caucasian**	**African**	**Asian**
	**(n = 62)**	**(n = 46)**	**(n = 10)**	**(n = 6)**	**(n = 536)**	**(n = 373)**	**(n = 81)**	**(n = 82)**
*Age(years)*	24.1 ± 3.7^1^	24.2 ± 4.0	23.8 ± 2.2	23.7 ± 4.2	37.5 ± 5.2^1^	37.8 ± 5.0	37.8 ± 5.3	36.1 ± 6.1
*BMI(kg/m*^*2*^*)*	21.0 ± 2.8^2^	20.6 ± 2.6	22.3 ± 2.8	21.5 ± 4.1	24.5 ± 5.1^2^	24.5 ± 5.3	26.8 ± 5.1	22.2 ± 3.1
*Estradiol(pg/mL)*	51.5 ± 26.1	52.5 ± 27.7	49.0 ± 19.4	46.2 ± 21.6	55.0 ± 37.8	57.0 ± 40.2	51.9 ± 34.5	48.8 ± 27.5
*FSH(mIU/mL)*	6.8 ± 2.9^3^	6.7 ± 3.0	6.6 ± 1.5	8.2 ± 2.8	12.3 ±10.1^3^	12.5 ± 10.2	13.8 ± 13.4	10.2 ± 4.1
*AMH(ng/mL)*	4.5 ± 2.9^4^	4.3 ± 3.0	5.7 ± 1.9	4.6 ± 3.7	1.7 ± 2.2^4^	1.6 ± 1.9^5^	2.0 ± 2.9	2.3 ± 2.4^5^
*Oocyte yield*	17.7 ± 7.7^6^	16.7 ± 6.6^7^	23.7 ± 10.4^7,8^	14.8 ± 6.4^8^	8.1 ± 6.7^6^	8.1 ± 6.8	7.9 ± 7.3	8.1 ± 5.9
*FMR1 n (%)*								
*norm*	33 (53.2)	26 (56.5)	4 (40.0)	3 (50.9)	315 (58.8)	218 (58.4)	41 (50.6)	56 (68.3)
*het-norm/high*	6 (9.7)	3 (6.5)	1 (10.0)	2 (33.3)	86 (16.0)	51 (13.7)	18 (22.2)	17 (20.7)
*het-norm/low*	19 (30.6)	14 (30.4)	5 (50.0)	0 (0.0)	107 (20.0)	83 (22.3)^9^	19 (23.5)^9^	5 (6.1)^9^
*hom*	4 (6.5)	3 (6.5)	0 (0.0)	1(16.7)	28 (5.2)	21 (5.6)	3 (3.7)	4 (4.9)
*Primary infertility diagnosis n (%)*								
*DOR*	N/A	N/A	N/A	N/A	279 (52.1)	199 (47.5)	38 (47.5)	42 (51.2)
*Male factor*	N/A	N/A	N/A	N/A	134 (25.0)	96 (25.7)	18 (22.5)	20 (24.4)
*Tubal Disease*	N/A	N/A	N/A	N/A	105 (19.6)	70 (16.7)	23 (25.3)	12 (13.6)
*Endometriosis*	N/A	N/A	N/A	N/A	27 (5.0)	21 (5.6)	1 (1.3)	5 (6.1)
*Uterine factors*	N/A	N/A	N/A	N/A	36 (6.7)	20 (5.3)	1 (1.2)	15 (18.8)
*PCOS*	N/A	N/A	N/A	N/A	39 (7.3)	27 (7.2)	6 (7.5)	6 (7.3)

Values are presented as means ± SD. Subscripts denote significant differences at P ≤ 0.05 (chi-square, t-tests and Holm-Sidak post hoc comparisons). ^1^ P = 0.001; ^2^ P = 0.001; ^3^ P = 0.001; ^4^ P = 0.001; ^5^ P = 0.007; ^6^ P = 0.001; ^7^ P = 0.008; ^8^ P = 0.022; ^9^ P = 0.012.

Once patients are diagnosed with DOR, they are pre-supplemented with micronized dehydroepiandrosterone (DHEA, 25 mg TID per os) for at least six weeks, prior to IVF cycle start. Ovarian stimulation involves a microdose agonist protocol and stimulation with 450–600 IU of gonadotropins daily, all given as an FSH product except for 150 IU, which is given as hMG [14].

Ovarian *FMR1* genotypes and sub-genotypes were determined by Southern blot hybridization and polymerase chain reaction (PCR), as previously reported [3,7,8]. In brief, based on a normal range of CGG_n=26–34_, patient were determined to have a normal (*norm*) genotype if both alleles were in normal range, as heterozygous (*het*) if one was outside normal range and as homozygous (*hom*) if both were outside normal range. *Het* and *hom* genotypes, depending on whether counts were above (*high*) or below (*low*) normal range, were then further sub-divided.

Statistical analysis was performed using SPSS version 18.0 (SPSS Inc., Chicago, IL). Chi-Square tests were used to compare proportions. Continuous variables were presented as mean and S.D., and were tested by student’s t-tests as well as analysis of variance. Post-hoc assessments were performed using the Holm-Sidak method. A P-value of < 0.05 was considered statistically significant.

This study underwent expedited IRB review since it only involved analysis of anonymized data from the center’s electronic research database. All of the center’s patients sign at initial visit an informed consent, which allows for use of medical record data for research purposes, as long as the patient’s anonymity and the medical record’s confidentiality are maintained. Both conditions were met here. Like the center’s clinical staff, the center’s research staff is in writing committed to confidentiality under federal HIPAA rules.

## Results

Table 1 summarizes patient characteristics for both patient populations studied. As the table demonstrates, oocytes donors were overall younger (P ≤ 0.001), had lower BMI (P ≤ 0.001), demonstrated lower FSH values (P ≤ 0.001), higher AMH levels (P ≤ 0.001) and higher oocyte yields (P ≤ 0.001). Total estradiol values did not differ between the two groups.

As Table 1 also demonstrates, donors as well as patients in each group, based on race, did not differ in age, BMI, estradiol and FSH. Based on race, donors also did not differ in AMH, though amongst infertility patients Asians demonstrated significantly higher AMH than Caucasians (P = 0.007). African donor demonstrated the highest AMH values; but they did not differ significantly from the other two races. In contrast, patients did not differ based on race, in oocyte yields, while donors did demonstrate significant differences between races, with African donors producing significantly more oocytes than Caucasian (P = 0.007) and Asians donors (P = 0.022).

**Figure 1:**
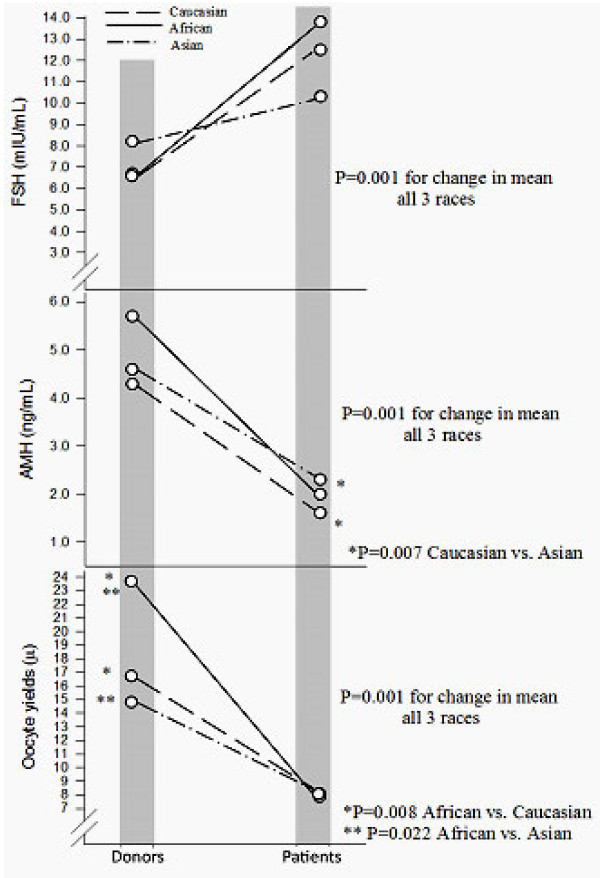
**Cross-sectional comparison between races in FOR parameters in oocyte donors and infertility patients, as function of change over time.** Means were significantly different between races for FSH, AMH and oocyte yields (all P = 0.001).

Figure 1 demonstrates how AMH, FSH and oocyte yields, depending on race/ethnicity, change at different ages. Changes in means between the two groups were significant for FSH (P = 0.001), AMH (P = 0.001) and oocyte yields (P = 0.001).

FSH did not differ either amongst donors or patients based on race. In Caucasian women mean FSH increased from 6.7 ± 3.0 mIU in young oocyte donors to 12.5 ± 10.2 mIU in infertility patients, representing an increase in mean level of 86.6 percent. African women increased from 6.6 ± 1.5 to 13.8 ± 13.4 mIU/mL, a 109.1 percent increase, while Asian women only increased from 8.2 ± 2.8 to 10.3 ± 4.2 mIU/mL, only a 25.6 percent increase.

AMH did significantly differ between Caucasian and Asian patients, with Asian women demonstrating higher levels (P = 0.007). AMH declined in Caucasians from 4.3 ± 3.0 ng/mL in donors to 1.6 ± 1.9 ng/mL in infertility patients, a 62.8 percent decline in mean, in Africans from 5.7 ± 1.9 ng/mL in donors to 2.0 ± 2.9 ng/mL in patients, a 64.9 percent decline and in Asians from 4.6 ± 3.8 ng/mL to only 2.3 ± 2.4 ng/mL, an only 50.0 percent decline in mean.

Finally, the most obvious race-based differences were seen in oocyte donors in regards to oocyte yields. African donors produced by far the largest oocyte numbers, significantly more than Caucasian (P = 0.008) and Asian donors (P = 0.022). Oocyte yields in Caucasians went from 16.7 ± 6.6 in donors to 8.1 ± 6.8 in infertility patients, a 51.5 percent decrease in mean, in Africans from 23.7 ± 10.4 to 7.9 ± 7.3, a 66.7 percent decline, and in Asians from 14.8 ± 6.4 to 8.1 ± 5.9, a 45.3 decline.

he percentage of *FMR1* genotypes and sub-genotypes differed significantly between races. Specifically, fewer Asian patients demonstrated the *het-norm/low FMR1* sub-genotypes than either Caucasian or African women (P = 0.012).

## Discussion

Different races are characterized by significant variances in reproductive performance. [4,5]. In association with IVF, women of African descent have been reported to have poorer IVF outcomes than Caucasians [15-17], though at least one study casts doubt on such a conclusion [6]. Similarly, Asian (usually Chinese) [17,18] and Hispanic [18,19] patients were also reported to demonstrate poorer IVF outcomes in comparison to Caucasian women.

We in this study excluded Hispanic patients if they were difficult to classify by race/ethnicity. Our center has been attempting to elucidate causes for observed outcome discrepancies between races/ethnicities for some time. When we noted that Asian/Chinese oocyte donors demonstrate lower mean FOR than their Caucasian and African counterparts, we, as we now know incorrectly, concluded that they suffered from a higher prevalence of premature ovarian aging [20]. We, however, since learned that Asian/Chinese women, simply, age ovaries differently than Caucasian and African women.

This became apparent when we investigated CGG_n_[21] and later ovarian *FMR1* genotypes and sub-genotypes in different races [3]. As it turned out, Chinese/Asian women demonstrated a distinctively different distribution of CGG_n_, also reflected in a distinctively different distribution of *FMR1* genotypes and sub-genotypes.

Specifically, Asian women demonstrated a much higher preponderance of *high* CGG counts (> 34) than Caucasian and African women, which, once ovarian *FMR1* genotypes and sub-genotypes had been defined, translated into a preponderance of the *het-norm/high* sub-genotype of the *FMR1* gene, associated with relative low FOR at young age, but disproportional preservation of FOR into older ages [22]. Such women, therefore, at younger ages, indeed, can be expected to have relatively lower pregnancy chances but will have a relatively more favorable outlook in comparison to other races at more advanced ages [22].

The opposing contrast to Chinese/Asian women is African females, who demonstrate a preponderance of low CGG counts on *FMR1*, and, therefore, a preponderance of the *het-norm/low* sub-genotype. Not surprisingly, Caucasians present with the most inhomogeneous distribution of ovarian *FMR1* genotypes and sub-genotypes [21,22].

Since ovarian *FMR1* genotypes and sub-genotypes are associated with specific ovarian aging patterns (i.e., declines in FOR) [7,8,22], differences in their respective distribution between races are associated with distinct ovarian aging patterns and, not surprisingly, also with distinct pregnancy outcomes in association with IVF [3,8,22].

We, therefore, concluded that the cross-sectional investigation of FOR in young egg donors and in older infertility patients of different races/ethnicities should offer another experimental model to investigate the association of ovarian *FMR1* genotypes and sub-genotypes in their potential impact on race differences in ovarian aging patterns. That genetic differences on ovarian aging exist has been suggested [1,2].

Our center serves a racially diverse group of infertility patients, with considerable minority representation of African and Asian patients, the latter mostly of Chinese ethnicity. For that reason the center’s egg donor pool has to represent a similar racial/ethnic background, and, indeed, does. Caucasian patients and donors, however, still dominate (Table 1). Especially in the smaller oocyte donor group, small numbers amongst minorities may, therefore, be responsible that only rather few differences were observed between races. At the same time, observed statistical differences, obviously, have to be viewed with caution until others confirm here presented data.

As Figure 1 probably best demonstrates, ovarian aging patterns differ significantly amongst all three here investigated races. They differ in all investigated parameters of FOR, FSH, AMH and oocyte yields (all P = 0.001). Differences between races, however, vary.

One conclusion, again reconfirmed in full agreement with earlier studies [3,21], the genetically most homogenous women are of Asian/Chinese descent. They start reproductive life with highest FSH levels and lowest oocyte yields in comparison to Caucasians and especially African women, as here demonstrated in young oocyte donors, and demonstrate the mildest increase in FSH and drop in AMH as well as oocyte yields with advancing age, as demonstrated in the infertile patient population.

As noted earlier, they, therefore, can be expected to have somewhat lower pregnancy chances at younger ages, possibly responsible for reports of lower IVF pregnancy rates in Asian/Chinese women [17,18]. They, however, should have an advantage over other races at older ages, where they still demonstrate higher FOR [22].

Their polar opposite are African women who start with lowest FSH, highest AMH and by far the highest oocyte yields amongst races; yet, as they age, they demonstrate the poorest FOR of all races, based on highest FSH and the largest declines in AMH and oocyte yields (Figure 1).

This picture, of course fits perfectly with the previously noted preponderance of the ovarian *het-norm/high* sub-genotype in Asian/Chinese and *het-norm/low* in African women [3,21]. The data also perfectly correlate with the significantly lower prevalence of the *het-norm/low* sub-genotype in Asian women (Table 1), though small donor numbers amongst minorities in this study do not allow for robust conclusions on this point.

The *het-norm/low FMR1* sub-genotype, here again demonstrated to be very prominently present amongst African women (50.0% amongst donors and 23.5% amongst patients), has been associated with a polycystic ovary-like ovarian phenotype, at young age associated with excessive follicle recruitment and, therefore, rapid depletion in FOR, leading to DOR at relatively young ages [8].

This is, indeed, exactly the ovarian aging pattern here primarily observed in women of African descent. This aging pattern, of course, very well explains widely reported poorer IVF pregnancy outcomes for women of African descent [15-17].

This study also adds support to recent reports in the literature questioning the sensitivity and specificity of AMH in defining FOR [9,11]. This is best demonstrated by the fact that, though African donors, by far, produced the highest oocyte yields, their AMH did not differ from other races. Similarly, Asian patients demonstrated significantly higher AMH than Caucasians; yet, oocyte yields were the same.

## Conclusions

Here presented data, therefore, reemphasize the evolving importance of the newly described ovarian genotypes and sub-genotypes of the *FMR1* gene in their association with typical phenotypical ovarian aging patterns. It, therefore, also does not surprise that they are statistically predictive of pregnancy chances with IVF [3,8].

The data also suggest that widely reported differences in IVF pregnancy chances between races, at least to a degree, are, likely, determined by *FMR1* genotypes and sub-genotypes. It, therefore, would appear important to integrate *FMR1* genotypes and sub-genotypes as co-variates in reproductive outcome studies. Indeed, *FMR1* genotyping can also be expected to increasingly enter clinical practice.

## Abbreviations

AMH: anti-Müllerian hormone; DHEA: dehydroepiandrosterone; DOR: diminished ovarian reserve; FOR: functional ovarian reserve; het: heterozygous; hMG: human menopausal gonadotropin; hom: homozygous; FSH: follicle stimulating hormone; *FMR1*: fragile X mental retardation 1; *norm*: normal; IVF: in vitro fertilization; IRB: Institutional Review Board; SD: standard deviation.

## Competing interests

N.G, A.W. and D.H.B. have in the past received research support, speakers’ honoraria and travel funds from various pharmaceutical and medical device companies, none, however, related to the subject of this paper. N.G and D.H.B, are listed as co-inventors of two awarded U.S. patents, claiming therapeutic benefits for DHEA, and potentially other androgens, in women with DOR. Both authors have other pending patent applications, regarding DHEA, and other androgens, and the *FMR1* gene’s effects on ovaries. N.G. owns shares in Fertility Nutraceuticals, LLC, a company that sells a DHEA product. N.G. and D.H.B. are receiving patent royalties from this company. N.G. is also the owner of The CHR, where this research was conducted.

## Authors’ contributions

NG and DHB participated in study design and data analysis. AK performed a majority of statistical analyses with contribution from DHB. NG drafted the manuscript. AW participated in study design. All authors reviewed and approved the final manuscript.
